# Associations of workforce characteristics, calendar time, and assessment modality with quality performance in family planning and post-abortion care services in Sierra Leone, 2019–2022

**DOI:** 10.1080/16549716.2026.2736342

**Published:** 2026-10-05

**Authors:** Felix Ikenna Onunkwor, Rose Aguolu

**Affiliations:** a Marie Stopes Sierra Leone, Freetown, Sierra Leone; b Department of Public Health, Texila American University, Georgetown, Guyana; c National Agency for the Control of AIDS (NACA), Abuja, Nigeria; d Federal Ministry of Health and Social Welfare, Abuja, Nigeria

**Keywords:** Longitudinal analysis, digital supervision, supportive supervision, reproductive health, health systems strengthening

## Abstract

**Background:**

Quality assessment in family planning (FP) and post-abortion care (PAC) programmes is shaped by workforce capability, programme context, and assessment modality. Longitudinal evaluations may misattribute quality-score differences if repeated measurements and temporal patterning are ignored.

**Objective:**

To examine associations of workforce characteristics, calendar period, service-delivery channel, and assessment modality with Quality Technical Assessment (QTA) performance in Sierra Leone, 2019–2022.

**Methods:**

We analysed 108 QTA records from 23 service delivery points using Gaussian generalised estimating equations with facility-level clustering and robust standard errors. Models included year, modality, channel, active staff count, and core-service competency coverage; full-time staffing and assessor identity were examined in sensitivity analyses. In 2021, all facilities received planned in-person and then remote assessments in a fixed programme-wide sequence.

**Results:**

Mean QTA scores increased descriptively from 89.8% (SD 7.4) in 2019 to 94.2% (SD 4.7) in 2022. In the primary model, 2020 scores were lower than 2019 (B = −5.15; 95% CI −9.99 to −0.32). Active staff count was inversely associated with QTA scores (B = −2.86; 95% CI −4.47 to −1.26). Remote assessment was associated with higher adjusted scores, but this cannot be interpreted causally because modality was confounded with year and assessment order.

**Conclusions:**

QTA performance varied across calendar periods and workforce structure. Longitudinal evaluations should account for within-facility correlation and modality timing before attributing quality differences to the workforce or assessment approach.

## Paper Context

- **Main findings**: Quality scores varied across calendar periods and staffing structure, while apparent differences between remote and in-person assessment were strongly entangled with when and in what sequence each approach was used.
- **Added knowledge**: This longitudinal programme evaluation shows how accounting for repeated facility measurements and the timing of assessment modality can materially change interpretations of workforce and quality-assessment associations.
- **Global health impact for policy and action**: Quality-assurance programmes should analyse repeated measurements longitudinally and interpret remote supervision within its implementation chronology, workforce context, and capacity for feedback and corrective action.

## Background

Family planning (FP) and post-abortion care (PAC) are core components of sexual and reproductive health, with service quality influencing informed choice, safe clinical practice, continuity of care, complication management, and client experience. In low-resource settings, maintaining quality across geographically dispersed service networks depends not only on the availability of services but also on a competent workforce, reliable clinical governance, adequate supplies, and quality-assurance systems capable of identifying and responding to gaps [1,2].

The global health workforce context makes these questions especially important. Updated estimates suggest a projected global shortage of approximately 10 million health workers by 2030, with the greatest deficits concentrated in lower-income settings [3]. For FP and PAC programmes operating through both fixed facilities and mobile or community-based outreach teams, staffing continuity, competency maintenance, and supervisory reach are persistent implementation challenges [4,5].

Quality assurance in FP and PAC has traditionally relied on in-person supervisory visits, during which trained assessors observe clinical practice, review documentation, verify environmental standards, and provide feedback [6,7]. Such approaches can be resource intensive where travel is difficult or expensive [4,5]. The COVID-19 pandemic disrupted conventional supervisory systems and accelerated the use of remote and digital approaches to maintain oversight when physical movement was restricted [8,9]. The period from 2019 to 2022 therefore provides a useful longitudinal window spanning pre-pandemic routine practice, acute disruption and remote adaptation, and subsequent recovery and consolidation.

A specific limitation of previous evaluations is that workforce-related confounding is often inadequately addressed when remote and in-person quality-assurance approaches are compared; differences attributed to assessment modality may instead reflect variation in staffing levels, provider competency, or other workforce characteristics that change across facilities and over time.

An important analytical problem is that assessment modality, workforce characteristics, and calendar time may change together. Evaluations that compare remote and in-person assessments without accounting for repeated observations within facilities, changing workforce composition, assessor effects, or the timing of modality deployment risk attributing temporal or contextual effects to modality itself. The evidence gap is therefore not simply whether remote and in-person assessments differ, but whether observed quality-score variation remains associated with workforce and modality after the longitudinal structure of programme data is modelled appropriately [10–13].

The repeated assessments followed defined programme procedures rather than ad hoc or performance-triggered scheduling. In 2021, all service delivery points underwent two planned assessments as part of a programme-wide remote-QTA pilot: an in-person assessment during March–April followed by a remote assessment during July–August, with a median interval of 144 days. The sequence was fixed across the network and was not determined by prior facility performance. The routine annual in-person assessment cycle was therefore maintained while the remote modality was piloted. In 2022, selected additional assessments were purposive and were undertaken for assessor-objectivity/calibration checks and, in at least one instance, performance follow-up.

Sierra Leone’s health system provides an important context for examining these relationships. The health workforce and supervisory system operate under persistent resource constraints, including shortages and uneven distribution of skilled personnel, geographically dispersed service delivery, infrastructure and referral limitations, and restricted capacity for frequent in-person supervision. These challenges occur within a health system that has experienced successive shocks, including civil conflict, the 2014–2016 Ebola epidemic and, more recently, COVID-19, each of which affected workforce capacity and service continuity. Within reproductive health services, these constraints are particularly relevant because maintaining technical quality in family planning and post-abortion care requires not only trained providers but also repeated competency assessment, supportive supervision, functioning referral systems, and reliable quality-assurance mechanisms [3,9,14,15].

Sierra Leone provides a relevant setting for this question. The programme evaluated here operated 23 service delivery points – 9 static Centres and 14 Outreach teams – across the 14 districts used in the programme’s operational structure during the study period. Centres were located mainly in district headquarters and urban or peri-urban settings, whereas Outreach teams provided last-mile services across rural and underserved areas. The principal service-scope difference was that Centres provided surgical PAC in addition to medical PAC, while Outreach teams provided medical PAC and referred clients requiring surgical management.

This study examined two primary questions: (1) how workforce characteristics – particularly active clinical staff count and core-service competency coverage – were associated with QTA performance over time; and (2) whether assessment modality remained associated with QTA performance after accounting for calendar period, service-delivery channel, repeated facility measurements, and selected workforce covariates. Incident-rate and qualitative analyses were retained as contextual components to support interpretation, not as causal tests of service complexity or modality equivalence.

## Methods

### Study design and setting

This longitudinal observational programme evaluation combined retrospective secondary analysis of routinely collected FP/PAC quality-assurance data from Sierra Leone for 2019–2022 with an explanatory qualitative component involving routine programme staff with direct operational experience of the quality-assurance process. The interval was selected deliberately to capture the pre-pandemic baseline (2019), the acute pandemic-disruption period in which physical supervisory travel was restricted (2020), and the subsequent adaptation and recovery period (2021–2022). The programme included 23 service delivery points: 9 static Centres and 14 community-based Outreach teams distributed across the 14 districts used by the programme during the study period.

The workforce represented the clinical service providers contributing to FP/PAC delivery at each service delivery point. The linked workforce database was aggregated at facility-year-channel level rather than individual professional-cadre level; consequently, this analysis evaluates workforce structure and competency coverage across the clinical team and does not estimate profession-specific effects. Core-service competency was defined using a standardised programme of competency assessment for counselling, implant insertion, and intrauterine device services.

Competency was classified using the programme’s three-level assessment system. Level 1 indicated that the provider completed all required steps correctly and was competent to provide the service independently, subject to regular reassessment. Level 2 indicated that all critical (‘bold’) steps were completed correctly but one or more other steps were missed; these providers were permitted to provide the service only under the direct supervision of a Level-1 competent clinical staff member until subsequently endorsed as fully competent. Level 3 indicated that one or more critical steps were missed; these providers were not permitted to provide the service independently and required further training by a competent trainer, either on-site or off-site. For this analysis, core-service competency coverage represented the proportion of relevant providers classified as Level 1 in counselling, implant insertion, and intrauterine device services.

Workforce composition differed by service-delivery channel. Outreach teams were composed almost entirely of State Enrolled Community Health Nurses (SECHNs), with one team including a registered nurse. Centre-based teams were more mixed and included Clinical Health Officers, midwives, SECHNs, and a smaller number of doctors who provided support to the channel. However, the linked workforce dataset available for analysis was aggregated at the service-delivery-point-year-channel level rather than by professional cadre; consequently, the present analysis evaluates workforce structure and competency coverage at team level and does not estimate profession-specific effects.

Quality assurance was implemented as a repeated programme cycle rather than as isolated annual assessments. The QTA findings were communicated to service-delivery teams and used to identify performance gaps, develop corrective action plans, guide supportive supervision and targeted training or mentoring, and inform subsequent follow-up assessments. Consequently, later assessment years represent cumulative exposure to successive quality-improvement cycles as well as broader programme and workforce changes over time [1,16,17].

The programme implemented a structured quality-improvement cycle alongside each QTA assessment. Following each visit, assessors produced a written feedback report identifying standards not met and specific corrective actions. These reports were shared with facility managers within a median of 1–2 working days. An action plan was developed with agreed responsibilities and timelines, and a follow-up supervisory visit was scheduled to verify implementation. Where competency gaps were identified, targeted on-the-job training was delivered at the point of care by Medical Development Team personnel, using a structured four-stage teaching approach. This cycle – assess, feedback, plan, train, and re-assess – was applied consistently across the study period and represents the programme’s primary mechanism for translating quality assessment into quality improvement. It provides important context for interpreting the temporal quality trends observed in this study, although the observational design cannot attribute improvement causally to any single component of the cycle.

### Quality technical assessment instrument and assessor standardisation

The QTA was a standardised programme checklist generating a continuous composite final score from 0 to 100. It covered six broad quality domains: clinical governance, clinical record audit, product quality, client-centred care, service delivery, and safety. Domain evidence included review of governance and incident-management records, commodities and storage, counselling and privacy, procedure-specific service standards, emergency readiness, and clinical documentation. Trained assessors applied common scoring guidance and standard operating procedures.

Seven Level-1 competent assessors conducted the assessments during the study period; five were MSI Master Trainers. Assessor eligibility required demonstrated competence in the service area assessed, assessor certification, a valid annual competency-assessor certificate, and completion of recognised assessor training. Assessors underwent standardisation and were periodically supervised or cross-checked. Where feasible, deployment outside an assessor’s routine operational area was used to reduce familiarity bias. In 2022, selected repeat assessments were also used for assessor-objectivity checks and training of newer assessors.

### Remote assessment procedures

Remote and in-person assessments used the same QTA domains, scoring criteria, and quality standards. Remote evidence was obtained predominantly through live audiovisual communication, supplemented where required by photographs, electronic document sharing, screen sharing, provider interviews, record review, and remote observation of clinical procedures. Areas that required visual confirmation were assessed remotely rather than omitted. For observed clinical procedures, video framing focused on the procedure and avoided identifying the client; the assessment process included an explanation of consent and documented client consent [11].

Client-centred and clinical-practice domains were therefore not based solely on provider self-report. Depending on the item, evidence could include live observation of provider–client interaction, review of documentation, verification of counselling and privacy processes, and corroborating records. Nevertheless, remote assessment can be vulnerable to selective presentation of evidence and social-desirability effects; these risks are addressed explicitly in the limitations.

Modality allocation was not random. In 2019, all usable assessments were in person; in 2020, 14 of 15 were remote; in 2021, we included both modalities; and in 2022, all usable assessments were in person. In 2021, where both modalities were implemented within the same network, in-person assessment was systematically preceded by remote assessment. The median interval between modalities was approximately 144 days (IQR 105–154). Modality coefficients were therefore interpreted as associations within this temporally patterned implementation, not as causal or equivalence estimates.

### Data sources and analytical dataset

Three programme data sources were linked using service-delivery-point identifiers, year, and channel: the QTA database, workforce competency/staffing records, and programme safety records. Workforce measures included number of active clinical staff, proportion of active clinical staff on full-time contract, and average core-service competency coverage. Recent training/support coverage showed no meaningful dispersion in the linked analytical sample and was not entered into the multivariable modelling.

The source QTA file contained 110 assessment records for 2019–2022. Two 2021 records had QTA Final Score coded 888, a predefined sentinel value for unusable/non-applicable final scores, and were excluded from analyses requiring the final score. The final analytical sample therefore comprised 108 usable QTA observations: 23 in 2019, 15 in 2020, 44 in 2021, and 26 in 2022. Because individual service delivery points were assessed repeatedly – and sometimes more than once within a year – the assessment record, rather than the facility-year, was the observation unit, with within-facility dependence handled in the longitudinal model.

### Statistical analysis

QTA Final Score was treated as a continuous outcome on its 0–100 scale. Descriptive statistics were examined by year, channel, modality, and assessor. Because several explanatory variables were non-normal or ordinal/binary and QTA scores showed upper-range compression, Spearman rank correlations were used for bivariate robustness analysis.

The primary longitudinal analysis used generalized estimating equations (GEE) with Gaussian distribution, identity link, exchangeable working correlation, and robust sandwich standard errors, clustering repeated observations by service delivery point. Calendar year was modelled categorically with 2019 as the reference category. The primary model included year, assessment modality, service-delivery channel, number of active clinical staff, and average core-service competency coverage. A prespecified sensitivity model additionally included the proportion of active clinical staff on a full-time contract.

Because only seven assessors contributed observations and assessment counts were markedly unequal between assessors, a crossed random-effects model was not considered sufficiently stable for the available sample. Instead, assessor identity was entered as a categorical fixed effect in the sensitivity GEE while retaining facility-level clustering. This directly assessed whether the principal associations were materially altered after accounting for assessor-related variation.

The overall calendar-year effect was assessed using a joint Wald test. Regression coefficients are reported with 95% confidence intervals and two-sided *p* values. A paired 2021 modality analysis was retained only as a descriptive sequencing check among facilities with usable scores under both modalities; because in-person assessment always preceded remote assessment, this comparison was not interpreted as a modality effect.

Incident rates per 1,000 couple-years of protection were available as annual channel-level aggregates. The Centre-versus-Outreach Mann–Whitney comparison therefore comprised eight channel-year observations (four annual Centre rates and four annual Outreach rates). This small ecological comparison was treated as contextual. Service complexity was not measured directly and was not entered as an explanatory variable. Descriptive and programming analyses were conducted in IBM SPSS Statistics; the revised longitudinal GEE and robustness analyses were independently verified using Python (statsmodels).

### Qualitative component

The qualitative strand was used as an explanatory contextual layer rather than as an independent causal test. Sources included open-ended questionnaire responses, one-to-one post-assessment discussions with seven assessors, and a Microsoft Teams focus group involving seven assessors and five District Family Planning Managers (12 participants). These qualitative data were collected directly from the participants rather than extracted from routine programme records. Participants were purposively selected on the basis of direct operational experience of remote and/or in-person quality assessment during the study period [18]. All seven Level-1 certified assessors and five District Family Planning Managers who were active during the study period were approached by the principal investigator through established programme channels. Participation was voluntary, and no incentive was offered. Informed verbal consent was obtained individually before participation in the qualitative activities, including open-ended questionnaire responses, one-to-one discussions, and the focus group. The approved study proposal explicitly provided for both verbal and written consent from clients and service providers under the ethical framework approved by the Sierra Leone Ethics and Scientific Review Committee. Verbal rather than written consent was considered appropriate and proportionate for these minimal-risk discussions of participants’ professional experience, particularly where interaction occurred remotely. Participants were informed of the purpose of the research, their right to decline or withdraw participation, and that their responses would be anonymised in analysis and reporting. For the one-to-one exploratory discussions and the focus group, the principal investigator documented verbal consent in the discussion schedule/protocol before participation. Immediately before the Microsoft Teams focus-group recording commenced, participants were informed that recording would begin and were given the opportunity not to proceed. Participants were also provided with the contact details of the Sierra Leone Ethics and Scientific Review Committee should they have concerns about the research or their participation. The broad prompts covered overall experience, what worked well, operational challenges, comparison with in-person assessment, ability to verify service quality and provider performance, concerns about reliability/confidentiality/completeness, contextual influences, lessons learned, and recommendations for future remote or hybrid models. The broad contextual validation prompts are provided in the separate Appendix 1 file.

Initial qualitative coding was undertaken collaboratively by the principal investigator and an MDT member (Octavia Johnson), with methodological support from the programme Research, Monitoring, and Evaluation lead. Codes combined inductive concepts emerging from participant accounts with pre-identified implementation-risk domains in the programme remote-assessment Terms of Reference. Felix Ikenna Onunkwor and Rose Aguolu subsequently reviewed the coding framework and preliminary themes; interpretive differences were discussed and resolved by consensus [19]. This process is described as collaborative coding with author review, not independent double-coding. The authors also acknowledge Dr Olaiya Paul Abiodun (National Open University of Nigeria) for methodological guidance and supervisory support during the development of this work.

Reflexivity was considered important because the principal investigator had a leadership role in the quality-assurance programme. To reduce over-attribution to programme experience, qualitative interpretations were grounded in participant accounts, reviewed by co-analysts and used to contextualise quantitative findings rather than to establish causal mechanisms.

### Ethics

This longitudinal observational programme evaluation combined retrospective secondary analysis of de-identified routine programme records and associated quality-assessment materials with primary qualitative information obtained from routine programme staff with direct operational experience of the quality-assurance process. Ethical approval covering the research was granted by the Sierra Leone Ethics and Scientific Review Committee, Directorate of Training and Research, Ministry of Health and Sanitation (Reference No. 008/09/2022, valid through 20 July 2023). The approved study proposal explicitly provided for verbal and written consent from clients and service providers. In client-facing remote-assessment activities, verbal client consent was documented on the client record form by indicating that the provider had obtained the consent. For the qualitative expert discussions and focus group, verbal consent was documented by the principal investigator in the discussion schedule/protocol before participation. No individually identifiable data were reported. The study was conducted in accordance with the Declaration of Helsinki.

## Results

### Analytical sample and assessment distribution

The final analytical dataset comprised 108 usable QTA assessments from 23 service delivery points. The mean QTA Final Score increased descriptively from 89.84 (SD 7.36) in 2019 and 90.40 (SD 5.91) in 2020 to 93.88 (SD 4.36) in 2021 and 94.23 (SD 4.73) in 2022. Scores were generally high: 52 of 108 assessments (48.1%) scored at least 95 and 2 reached 100, indicating some upper-range compression.

Assessment modality was strongly patterned by calendar year. The 2019 records were entirely in person (23/23), 14 of 15 assessments in 2020 were remote, 2021 contained 22 usable in-person and 22 usable remote assessments, and all 26 usable 2022 assessments were in person. Across the full sample, there were 72 in-person and 36 remote assessments. Channel distribution was 43 Centre assessments and 65 Outreach assessments. The seven assessors contributed unequally: Octavia Johnson 28 assessments, Elizabeth Younger 27, Mamie Conteh 22, Hassan Shaw 15, Felix Ikenna 10, Monica Greene 4, and Diaminatou Soe 2. A transcription error in the original submission reported these counts as 43 in-person and 65 remote (which were Centre and Outreach counts); this has been corrected. The assessment distribution and full-time staffing summary are presented in Table 1.

**Table 1.** Distribution of usable QTA assessments and full-time staffing by year, service-delivery channel, and assessment modality (*N* = 108).

Year	Total	Centre	Outreach	In-person	Remote	Full-time staff, mean % (SD)
2019	23	9	14	23	0	80.4 (29.2)
2020	15	5	10	1	14	93.3 (17.6)
2021	44	17	27	22	22	93.5 (17.2)
2022	26	12	14	26	0	85.0 (23.5)
Total	108	43	65	72	36	87.0 (23.3)

### Bivariate robustness analysis

Spearman analysis showed modest associations rather than large effects. The QTA score was negatively associated with active clinical staff count (ρ = −0.258, *p* = .007) and positively associated with core-service competency coverage (ρ = 0.258, *p* = .007) and calendar year (ρ = 0.277, *p* = .004). Assessment modality (ρ = 0.179, *p* = .064), service-delivery channel (ρ = 0.074, *p* = .446), and full-time staff proportion (ρ = −0.113, *p* = .246) were not significantly associated with QTA scores in the bivariate rank analysis. The bivariate Spearman associations are presented in Table 2.

**Table 2.** Spearman associations with QTA final score (*N* = 108).

Variable	Coding / scale	Spearman ρ	*p* value
Calendar year	2019–2022	0.277	0.004
Assessment modality	In-person = 1; Remote = 2	0.179	0.064
Service-delivery channel	Centre = 1; Outreach = 2	0.074	0.446
Active clinical staff	Count	−0.258	0.007
Core-service competency coverage	0–100%	0.258	0.007
Full-time staff proportion	0–100%	−0.113	0.246

### Facility-clustered GEE

The primary GEE accounted for repeated observations within the 23 service delivery points. The joint categorical year effect was statistically significant (Wald χ^2^ = 12.69, df = 3, *p* = .005). Relative to 2019, adjusted QTA scores were lower in 2020 (B = −5.15, 95% CI −9.99 to −0.32, *p* = .037), while the 2021 (B = 0.08, 95% CI −3.34 to 3.51, *p* = .963) and 2022 (B = 2.39, 95% CI −1.51 to 6.30, *p* = .229) contrasts were not statistically significant after simultaneous adjustment.

Active clinical staff count was negatively associated with QTA Final Score (B = −2.86 per additional staff member, 95% CI −4.47 to −1.26, *p* < .001). Core-service competency coverage was positively signed but not statistically significant in the primary model (B = 0.056 per percentage point, 95% CI −0.028 to 0.140, *p* = .190). Channel was not independently associated with QTA scores (Outreach versus Centre: B = −0.31, 95% CI −2.34 to 1.71, *p* = .762).

Remote assessment was associated with a higher adjusted QTA score (B = 4.27, 95% CI 2.37 to 6.16, *p* < .001). This estimate must be interpreted in light of the strong calendar-time and sequence confounding described above. The sensitivity model adding full-time staffing gave materially similar estimates for remote assessment (B = 4.25, 95% CI 2.35 to 6.15, *p* < .001) and staff count (B = −2.63, 95% CI −4.27 to −0.99, *p* = .002); competency coverage reached borderline significance (B = 0.085, 95% CI 0.001 to 0.170, *p* = .048), whereas full-time staff proportion was not significant (B = −0.040, 95% CI −0.086 to 0.006, *p* = .089).

In an assessor-adjusted GEE sensitivity analysis, the principal associations were not materially changed: remote assessment remained positively associated with QTA scores (B = 3.90, 95% CI 1.48 to 6.33, *p* = .002), and staff count remained negatively associated (B = −2.59, 95% CI −3.86 to −1.32, *p* < .001). Competency coverage remained non-significant (B = 0.057, *p* = .157). These results indicate that assessor variation did not explain the principal staff-count or modality associations. The primary and full-time-staff sensitivity GEE estimates are presented in Table 3.

**Table 3.** Generalised estimating equation models predicting QTA final score (*N* = 108; 23 facility clusters).

Predictor	Primary GEE B (95% CI)	*p*	Full-time sensitivity B (95% CI)	*p*
2020 vs 2019	−5.15 (−9.99, −0.32)	0.037	−5.27 (−9.87, −0.67)	0.025
2021 vs 2019	0.08 (−3.34, 3.51)	0.963	0.02 (−3.38, 3.43)	0.989
2022 vs 2019	2.39 (−1.51, 6.30)	0.229	1.95 (−1.80, 5.70)	0.308
Remote vs in-person	4.27 (2.37, 6.16)	<0.001	4.25 (2.35, 6.15)	<0.001
Outreach vs Centre	−0.31 (−2.34, 1.71)	0.762	−0.31 (−2.36, 1.73)	0.764
Active clinical staff	−2.86 (−4.47, −1.26)	<0.001	−2.63 (−4.27, −0.99)	0.002
Core-service competency (%)	0.056 (−0.028, 0.140)	0.190	0.085 (0.001, 0.170)	0.048
Full-time staff proportion (%)	—	—	−0.040 (−0.086, 0.006)	0.089

Note: Gaussian GEE with identity link, exchangeable working correlation, robust standard errors, and clustering by service delivery point. Calendar year was categorical with 2019 as reference. Overall year effect in the primary model: Wald χ^2^ = 12.69, df = 3, p = .005. CI = confidence interval.

### 2021 sequencing check

Among 21 service delivery points with usable QTA Final Scores under both modalities in 2021, mean in-person QTA score was 91.52 and mean remote QTA score was 96.03, a mean within-facility difference of 4.51 points (paired *t* = 3.78, df = 20, *p* = .001). However, in-person assessment systematically occurred first and remote assessment later. The higher later score may reflect corrective action after the first assessment, programme maturation, elapsed time, or other period effects. It therefore cannot be interpreted as evidence that remote assessment produces superior measurement or quality outcomes.

### Incident rates by service-delivery channel

The channel-level incident analysis used four annual Centre rates and four annual Outreach rates (*N* = 8 channel-year observations). Centre-based services had higher total incident-rate ranks than Outreach services (U = 0.00, Z = −2.31, *p* = .021; *r* = −0.82). In 2021, for example, the reported clinical incident rate was 0.28 per 1,000 CYP in Centres and 0.01 per 1,000 CYP in Outreach.

The difference should not be attributed directly to case complexity because case-mix severity was not measured in the analytical dataset. Programme context nevertheless differed: Centres provided both medical and surgical PAC, whereas Outreach teams provided medical PAC and referred clients requiring surgical management. The incident finding is therefore presented as an ecological channel-level difference requiring risk-adjusted follow-up, not as evidence of lower quality in Centres.

### Qualitative contextual findings

Qualitative findings were organised into three themes relevant to the quantitative interpretation. First, participants described remote assessment as flexible and capable of extending supervisory reach. One participant noted, ‘It’s not dependent on availability of clients on a specific assessment day … it presents an opportunity to observe services with low caseload.’ This illustrates perceived operational flexibility rather than proof of superior quality measurement.

Second, participants consistently favoured complementarity rather than a wholesale replacement of in-person assessment. Statements included, ‘It can be used to complement in-person assessments’ and ‘It is flexible and can be used as a hybrid to complement in-person assessment.’ These accounts support a programme preference for hybrid supervision but do not establish comparative effectiveness.

Third, connectivity, digital literacy, and document preparation were recurrent implementation prerequisites. Participants recommended, ‘Invest in internet boosters for specific teams,’ noted that ‘Teams are not computer literate,’ and advised that ‘All QA files and documents [should] be sent to the assessor before assessment.’ These findings indicate that remote supervision depended on infrastructure, preparation, and staff capacity to act on assessment requirements [12].

## Discussion

This revised longitudinal analysis changes the interpretation of the original manuscript in an important way. After repeated observations within service delivery points were handled using GEE, quality performance remained associated with the calendar period, but calendar year was not the only relevant factor. Active clinical staff count was independently and negatively associated with QTA score, while competency coverage showed a positive but model-sensitive association. Remote assessments also had higher adjusted scores, but the programme’s modality allocation was so strongly patterned by time and assessment sequence that the coefficient cannot be interpreted as a causal modality effect.

The year results should therefore be understood as temporal contrasts rather than evidence that calendar time itself caused the programme improvement. The descriptive annual means increased over the 4 years, yet the adjusted model showed a lower 2020 contrast relative to 2019 and no independent 2021 or 2022 contrast after simultaneous adjustment. Calendar periods may capture multiple time-varying influences: which facilities were assessed, pandemic travel restrictions, programme maturation, corrective action following prior QTAs, training and mentoring, staffing changes, assessor deployment, and other unmeasured organisational factors. Repeated quality-assessment cycles included feedback, action planning, and follow-up, making cumulative learning a plausible mechanism, but the observational design cannot partition the contribution of each mechanism.

The COVID-19 period is therefore best described as a context of temporal change rather than a natural experiment demonstrating pandemic effects. Assessment volume fell from 23 records in 2019 to 15 in 2020 because of COVID-19-related travel restrictions, and the Services Score decreased from 97.63% to 93.68% before recovering in 2021–2022. These are pandemic-period patterns without a counterfactual. Importantly, facilities not assessed in 2020 did not show evidence of materially different 2019 baseline QTA performance from those that were assessed, which reduces – but does not eliminate – concern about quality-driven selection into 2020 assessment.

The negative association between staff count and QTA score should not be interpreted to mean that additional staff reduce quality. Staff count may proxy service volume, organisational complexity, turnover, workload, or the difficulty of maintaining consistent standards across a larger team. These factors were not measured directly, so this explanation remains a hypothesis. The modest bivariate correlation also reinforces the need to distinguish statistical significance from effect magnitude.

Competency coverage warrants similarly cautious interpretation. Its positive bivariate association was modest, and it was not significant in the primary GEE, although it became borderline significant after adjustment for full-time staffing. The appropriate conclusion is therefore that competency may contribute to quality performance but its independent association is sensitive to model specification. The data do not justify claims that competency investment would necessarily produce larger quality gains than changes in assessment modality.

The remote-assessment finding requires particular restraint. A positive adjusted remote coefficient does not show that remote QTA is superior. Remote assessment occurred almost exclusively during 2020 and the later part of 2021, and in 2021, every in-person assessment preceded the remote assessment by a substantial interval. The descriptive paired difference is therefore equally compatible with temporal improvement, corrective action after the first QTA, or sequence effects. The study was not designed as an equivalence, non-inferiority, or randomised modality trial. Its contribution is instead to show why modality comparisons in routine programme data must be interpreted within their implementation chronology.

The detailed remote-QTA procedures nevertheless demonstrate that the remote approach was more than provider self-report. Live video, record review, document sharing, photographs, screen sharing, provider interviews, and remote procedure observation were used within the same standardised QTA framework. This procedural continuity supports the plausibility of remote monitoring, while qualitative evidence identifies prerequisites for credible use: digital connectivity, staff familiarity with assessment standards, organised documentation, consent and confidentiality safeguards, and mechanisms to respond to identified gaps. These are implementation conditions, not quantitatively proven effect modifiers [20,21].

The incident analysis remains useful only as contextual evidence. The significant Centre–Outreach rank difference was based on eight annual channel-level observations. Centres had a broader service scope and provided surgical PAC, but because client-level severity and case mix were not measured, the higher incident rates cannot be statistically attributed to complexity. Future safety analyses should use facility- or case-level denominators and risk adjustment.

This study has several strengths: a four-year programme census across 23 service delivery points; linkage of quality, workforce, and safety records; explicit modelling of within-facility correlation; assessor-adjusted sensitivity analysis; and qualitative contextualisation of how remote assessment functioned operationally. The revised analysis also makes transparent the two excluded sentinel-coded outcomes and the highly patterned distribution of modality over time.

Limitations include the observational single-programme design, non-random modality allocation, unequal numbers of assessments by year and assessor, multiple assessments within some facility-years, residual confounding, and the inability to estimate profession-specific workforce effects from facility-level staffing summaries. The QTA scores were generally high, with upper-range compression that may reduce sensitivity to smaller associations. The qualitative strand included programme personnel and was partly analysed by investigators involved in programme implementation, creating potential positionality bias despite collaborative coding and author review. Remote assessment may also be susceptible to selective evidence presentation or social-desirability effects. Finally, incident analysis was ecological and service complexity was not directly measured.

## Conclusions

This longitudinal programme evaluation highlights workforce structure and temporal context as important considerations in interpreting quality-assurance performance in FP/PAC services. The persistent association between staffing structure and QTA performance, together with the model-sensitive association of competency coverage, indicates that assessment modality should be considered within the broader workforce and quality-improvement system rather than in isolation. Programmes considering remote or hybrid assessment should therefore pair modality decisions with investments in provider competence, stable staffing, corrective-action systems, and mechanisms for follow-up and learning.

The apparent adjusted advantage of remote assessment should not be interpreted as evidence of superiority or equivalence because modality allocation was non-random, strongly patterned by calendar time, and fixed in sequence during the only year in which both modalities were widely used. The broader lesson is methodological and programmatic: longitudinal evaluations of supervisory systems should model within-site correlation, assessor variation, and the chronology of implementation before assigning effects to workforce characteristics or assessment modality. Remote assessment may remain a useful component of hybrid quality assurance when evidence verification, digital readiness, consent, feedback, and follow-up systems are in place.

## Supplementary Material

GHA_266593644_Appendix_1_Qualitative_Contextual_Validation_Prompts.docx

Suplementary File1_STROBE Checklist.docx

## Data Availability

The de-identified quantitative dataset supporting the findings of this study is publicly available on Zenodo: Onunkwor FI. Merged QTA and Workforce Competency Dataset – FP/PAC Quality Assurance Programme, Sierra Leone (2019–2022), Version 1.0. Zenodo. https://doi.org/10.5281/zenodo.21322808. No individually identifiable data are included.

## Open scholarship

This article has earned the Center for Open Science badge for Open Data. The data are openly accessible at https://doi.org/10.5281/zenodo.21322808
