# Supplementary material for: Associations of workforce characteristics, calendar time, and assessment modality with quality performance in family planning and post-abortion care services in Sierra Leone, 2019–2022
Source: Glob Health Action. 2026 Oct 5;19(1):2736342. doi: 10.1080/16549716.2026.2736342 (PMC13644452; doi:10.1080/16549716.2026.2736342)
Supplement: Supplementary file 1 [file ZGHA_A_2736342_SM6167.docx]

**STROBE Checklist — Observational Studies (Longitudinal / Cohort)**

*Manuscript: Temporal Quality Improvement, Workforce Capability, and Supervisory Modality in FP/PAC Services: A Four-Year Longitudinal Programme Evaluation in Sierra Leone (2019–2022)*

Journal: Global Health Action | Study design: Longitudinal observational programme evaluation

| **Item** | **Recommendation** | **Location in Manuscript** |
| --- | --- | --- |
| **TITLE AND ABSTRACT** | | |
| **1** | (a) Indicate the study's design in the title or abstract. (b) Provide an informative, balanced summary of what was done and found. | Title states 'Four-Year Longitudinal Programme Evaluation'; structured abstract (Background, Objective, Methods, Results, Conclusions) ✅ |
| **INTRODUCTION** | | |
| **2 — Background/rationale** | Explain the scientific background and rationale. | Background section, paragraphs 1–6 ✅ |
| **3 — Objectives** | State specific objectives and any prespecified hypotheses. | Background, final paragraph — two research questions stated ✅ |
| **METHODS** | | |
| **4 — Study design** | Present key elements of study design early in the paper. | Methods — Study Design and Setting: longitudinal observational design ✅ |
| **5 — Setting** | Describe setting, locations, and relevant dates. | Methods — 23 service delivery points, 14 districts, Sierra Leone, 2019–2022 ✅ |
| **6 — Participants** | Give eligibility criteria and sources/methods of selection. | Methods — Data Sources: complete programme census; 108 facility-channel-year records ✅ |
| **7 — Variables** | Clearly define all outcomes, exposures, predictors, potential confounders. | Methods — Data Sources: QTA final score outcome; competency, channel, year, modality predictors ✅ |
| **8 — Data sources/measurement** | Give sources of data and details of assessment methods. | Methods — Data Sources and Analytical Dataset: three linked programme data sources described ✅ |
| **9 — Bias** | Describe efforts to address potential sources of bias. | Methods — missing values (888/999) excluded; year covariate for temporal confounding; Limitations ✅ |
| **10 — Study size** | Explain how the study size was arrived at. | Methods — complete programme census; N = 108 ✅ |
| **11 — Quantitative variables** | Explain how quantitative variables were handled. | Methods — Statistical Analysis: continuous variables; year centred at 2020; coding stated ✅ |
| **12 — Statistical methods** | Describe all statistical methods including control for confounding. | Methods — Pearson correlation, multivariable linear regression with year and channel covariates, Mann–Whitney U; SPSS ✅ |
| **RESULTS** | | |
| **13 — Participants** | Report numbers at each stage of the study. | Results — Sample Characteristics: 108 records; year and channel distribution ✅ |
| **14 — Descriptive data** | Give characteristics of study participants. | Results — Sample Characteristics; Table 1 correlation matrix ✅ |
| **15 — Outcome data** | Report numbers of outcome events or summary measures. | Results — QTA scores by year, channel, and modality reported ✅ |
| **16 — Main results** | Give unadjusted and confounder-adjusted estimates with precision (95% CI). | Results — Table 2 adjusted models; B, 95% CI, p for all predictors; R², adjusted R² ✅ |
| **17 — Other analyses** | Report other analyses — subgroups, sensitivity analyses. | Results — sensitivity model (Model 2); incident rates by channel; qualitative evidence ✅ |
| **DISCUSSION** | | |
| **18 — Key results** | Summarise key results with reference to objectives. | Discussion, opening paragraph ✅ |
| **19 — Limitations** | Discuss limitations, sources of bias, direction and magnitude. | Strengths and Limitations — COVID-19 period effects and study limitations discussed explicitly ✅ |
| **20 — Interpretation** | Give cautious overall interpretation of results. | Discussion — temporal trend interpretation; causal language appropriately qualified ✅ |
| **21 — Generalisability** | Discuss generalisability (external validity). | Limitations — single programme context; LMIC programme relevance noted ✅ |
| **OTHER INFORMATION** | | |
| **22 — Funding** | Give source of funding and role of funders. | Funding information (end materials) — no external funding ✅ |

*Note. All 22 STROBE items are addressed in the manuscript. Checklist provided as a separate file alongside the manuscript in accordance with Global Health Action requirements.*
