# Supplementary material for: Associations of workforce characteristics, calendar time, and assessment modality with quality performance in family planning and post-abortion care services in Sierra Leone, 2019–2022
Source: Glob Health Action. 2026 Oct 5;19(1):2736342. doi: 10.1080/16549716.2026.2736342 (PMC13644452; doi:10.1080/16549716.2026.2736342)
Supplement: Supplementary file 2 [file ZGHA_A_2736342_SM6168.docx]

**Appendix 1. Qualitative contextual validation prompts**

Manuscript ID: 266593644

Associations of workforce characteristics, calendar time, and assessment modality with quality performance in family planning and post-abortion care services in Sierra Leone, 2019–2022

The following broad prompts guided the contextual validation discussions and open-ended qualitative component:

1. What was your overall experience with the remote quality assessment process?

2. What aspects of the remote assessment process worked well?

3. What challenges were encountered during remote assessment, including data sharing, internet connectivity, documentation, audio/video quality, or consent processes?

4. How did the remote assessment process compare with previous in-person assessment experience?

5. To what extent did the remote process allow assessors to verify service quality, client experience, and provider performance?

6. Were there any concerns about reliability, completeness, confidentiality, or interpretation of assessment findings?

7. What contextual factors influenced the success or limitations of remote assessment?

8. What lessons were learned for future remote or hybrid quality assessment models?

9. What recommendations would improve remote quality assessment in similar programme settings?
